# Breakfast patterns among low-income, ethnically-diverse 4^th^-6^th^ grade children in an urban area

**DOI:** 10.1186/1471-2458-14-604

**Published:** 2014-06-14

**Authors:** Hannah G Lawman, Heather M Polonsky, Stephanie S Vander Veur, Michelle L Abel, Sandy Sherman, Katherine W Bauer, Tim Sanders, Jennifer O Fisher, Lisa Bailey-Davis, Janet Ng, Gretchen Van Wye, Gary D Foster

**Affiliations:** 1 Center for Obesity Research and Education, Temple University School of Medicine, 3223 N. Broad Street suite 175, Philadelphia, PA 19140, USA; 2 The Food Trust, Philadelphia, USA; 3 Center for Health Research, Geisinger Health System, Danville, USA; 4 New York City Department of Health and Hygiene, New York, USA

**Keywords:** Childhood obesity, Minority health, Dietary intake

## Abstract

**Background:**

Increasing school breakfast participation has been advocated as a method to prevent childhood obesity. However, little is known about children’s breakfast patterns outside of school (e.g., home, corner store). Policies that increase school breakfast participation without an understanding of children’s breakfast habits outside of school may result in children consuming multiple breakfasts and may undermine efforts to prevent obesity. The aim of the current study was to describe morning food and drink consumption patterns among low-income, urban children and their associations with relative weight.

**Methods:**

A cross-sectional analysis was conducted of data obtained from 651 4^th^-6^th^ graders (51.7% female, 61.2% African American, 10.7 years) in 2012. Students completed surveys at school that included all foods eaten and their locations that morning. Height and weight were measured by trained research staff.

**Results:**

On the day surveyed, 12.4% of youth reported not eating breakfast, 49.8% reported eating one breakfast, 25.5% reported eating two breakfasts, and 12.3% reported eating three or more breakfasts. The number of breakfasts consumed and BMI percentile showed a significant curvilinear relationship, with higher mean BMI percentiles observed among children who did not consume any breakfast and those who consumed ≥ 3 breakfasts. Sixth graders were significantly less likely to have consumed breakfast compared to younger children. A greater proportion of obese youth had no breakfast (18.0%) compared to healthy weight (10.1%) and overweight youth (10.7%, p = .01).

**Conclusions:**

When promoting school breakfast, policies will need to be mindful of both over- and under-consumption to effectively address childhood obesity and food insecurity.

**Clinical trial registration:**

NCT01924130 from http://clinicaltrials.gov/.

## Background

The documented benefits of children’s regular intake of breakfast include increased concentration and improved academic performance and behavior [1]. Thus, increasing participation in the national School Breakfast Program (SBP) is a common goal of federal efforts such as End Hunger in America and the Healthy, Hunger-Free Kids Law [2,3], state-level initiatives, and school districts [4,5]. Among schools that offer the National School Lunch Program, the number that also participate in the SBP has grown from 48.8% in 1990 to 91.2% in 2011 [4]. However, only half (50.4%) of low-income children that participate in the National School Lunch Program also participate in the SBP [4]. To encourage students’ participation in school breakfast, school boards in major cities including Chicago, Houston, Memphis, Philadelphia, and Washington D.C. have adopted programs to offer breakfast in the classroom [5]. This is seen as a way to combat stigma associated with school breakfast participation [6], to address logistical challenges with breakfast served before school, and to fight food insecurity [7] and childhood obesity [5].

Providing breakfast at school for children who would not otherwise have one is valuable, especially to address food insecurity and improve academic performance. There are less data to support the notion that school breakfast has any impact on childhood obesity [1,8]. Public concerns have been raised over the possibility that breakfast in the classroom could unintentionally increase energy intake and undermine obesity prevention efforts among children who are already consuming one or more breakfasts outside of school [9]. One recent study found that 30.0% - 51.1% of 3^rd^ -5^th^ graders in New York City reported early morning eating in multiple locations including home, school, and corner stores (also known as “bodegas”) [10]. Only two studies with elementary aged children have examined the patterns of breakfast consumption across multiple locations [10,11]. However, the relationship between number and locations of breakfasts and relative weight were not assessed.

Previous research on breakfast consumption has focused on comparing breakfast consumption with no breakfast consumption rather than the patterns and quality of breakfast consumed [1]. Studies have examined a variety of breakfast contexts independently including breakfast prepared at home versus away from home [12], school breakfast consumption across the year [13], and breakfast that was self-prepared or consumed in the presence of others [14]. Other influences including mode of transportation to school and previous night’s dinner consumption may affect breakfast consumption patterns. Children who do not usually eat breakfast have been shown to be less likely to have had regular family dinners [15]. Collectively, these studies are limited in their reliance on comparing breakfast consumption with no breakfast consumption and have not considered multiple breakfasts.

Given the importance of understanding the breakfast habits of low socio-economic status youth and their potential impact on obesity risk, the purpose of the current study was to 1) assess breakfast patterns among 4^th^-6^th^ graders in an urban public school district and 2) to assess the relation between those patterns and measured relative weight.

## Methods

### Schools

Participants were recruited from three K-8 schools in Philadelphia that were participating in a pilot study to assess the feasibility of an intervention to promote eating one healthy breakfast. Schools were eligible if they met the following inclusion criteria: 1) at least 50% of students qualified for free/reduced price lunch, 2) served children in kindergarten - 8^th^ grade, 3) had no existing classroom breakfast feeding program (or were willing to give up classroom breakfast feeding), and 4) received nutrition education programming from The Food Trust, a community partner responsible for developing and implementing intervention curriculum. Eligible schools (n = 31) were matched on school size and race/ethnicity composition, and 6 schools were invited to participate. Four schools agreed, but one school dropped before the trial began. Eligible and participating schools were similar to other schools in the district with respect to percent minority, size, and percent qualifying for free and reduced lunch. The mean ± SD percent eligibility for free- or reduced-price meals for the three schools was 94.8 ± 1.6. All three schools provided all students with access to free breakfast in the cafeteria before school hours. All data for the current study were collected between October and November 2012 before any intervention had occurred. The study was approved by the Office of Research and Evaluation at the School District of Philadelphia and the Institutional Review Board at Temple University.

### Participants

Inclusion criteria for students in each of the three schools were a) enrolled in 4^th^-6^th^ grade, b) did not have a developmental disorder affecting their ability to understand the survey, and c) returned the consent and assent forms. Special dietary needs were not assessed. Parental consent and child assent forms were sent home with all 4^th^-6^th^ grade students, and students were asked to return signed forms indicating whether their parent approved or disapproved of their participation in the study. Among 1,047 eligible students, parental consent and child assent was obtained for 678 students (64.8%). Among those, 27 were removed for analyses (7 ineligible due to inability to read and understand the survey, 15 transferred schools before data collection was conducted, 4 were repeatedly absent, and 1 survey was incomplete) leaving a final sample of 651 4^th^-6^th^ grade students.

### Measures

All measures were obtained in the fall of 2012 in the morning after the school cafeteria breakfast was offered but prior to students’ scheduled school lunches. Children’s race, sex, month and year of birth, and grade level were obtained from the schools. Children’s race, as categorized by the school district based on parent self-report, was African American, Hispanic, Caucasian, Asian, or Other.

#### Weight and height

Trained research staff used standard protocols for height and weight measurements with portable stadiometers (SECA 217) and scales (SECA 869). Youth were instructed to remove shoes, any extra layers of clothing, and all items from pockets for measures. Height and weight were measured by taking 2 measurements required to be within 1 cm and .2 kg, respectively, or a third measure was taken and the two within the specified range were averaged. Inflexible hairstyles (e.g., braids) were measured and subtracted from overall height. Body Mass Index (BMI) and BMI z-scores and percentiles based on age and gender were calculated for each student based on CDC 2000 growth charts [16]. Weight status category was defined based on BMI percentile: underweight (<5^th^ percentile); healthy (≥5^th^ and <85^th^ percentile); overweight (≥85^th^ and < 95^th^ percentile); obese (≥100% to ≤ 120% of the 95^th^ percentile), and severely obese (≥120% of the 95^th^ percentile) [16,17].

#### Breakfast patterns survey

A questionnaire designed by the New York City Department of Health and Mental Hygiene and previously tested in same-aged school children in Philadelphia was used to measure morning food and drink patterns [10,18]. Participants were asked to report whether they ate or drank anything that morning (yes/no) from 1 of 4 locations: home, corner store, school cafeteria, or school classroom. Children were asked to report anything they had eaten or drank and were not directed about inclusion or exclusion of food items based on portion size. For each location, where a student reported eating or drinking something, students marked the items they ate/drank from a list of 19 food and drink categories (category list replicated in results table). These food and drink categories were based on common foods seen in school menus identified in previous work in the Philadelphia [19] and New York [10] areas.

For the purposes of the current study, “breakfast” was defined as having consumed any caloric food or beverage at a specified location. This definition was consistent with a previous study that defined breakfast as “consumption of any food or beverage other than plain water” [11]. Thus, if students reported any caloric food or drink items at a location it was considered a breakfast. Items included both traditional (e.g., eggs, waffles, bacon, yogurt) and non-traditional (e.g., chips, candy, soda) items. Breakfast surveys were completed before 10:30 am for 62% of the sample, between 10:30 and 11:45 am for 34% of the sample, and between 11:45 am and 12:10 pm for 4% of the sample. In all cases, breakfast surveys were completed before the student’s scheduled lunch period, and no schools offered snacks before school lunch. Students were instructed to categorize foods according to where they were received/purchased not where they were consumed (e.g., received apple at home and ate it at school). Therefore, data relate to where breakfast items were originally obtained rather than where they were ultimately consumed. Students, however, were instructed to only report items that they had already consumed that morning and to not report items that had been purchased but not yet consumed.

Students who reported not eating or drinking anything were asked to complete the question, “Please tell us why you didn’t eat or drink anything yet today? Please check all that apply.” and response options included “not hungry,” “not enough time,” “no food at home,” “No food I like,” and “No money to buy food”. The survey also assessed the consumption of dinner the evening before (yes/no) and whether students walked (yes/no) or were driven to school (yes/no).

### Data analysis

To assess morning food and drink consumption patterns, descriptive statistics examined food types and foods obtained across locations. Separate linear regressions were used to examine associations of race, grade, sex, relative weight (BMI percentile, BMI z-score, or prevalence of obesity), previous night’s dinner consumption, and method of transportation to school with number of breakfast consumed. Separate logistic regressions were used to examine associations of race, grade, sex, relative weight (BMI percentile, BMI z-score, or prevalence of obesity), previous night’s dinner consumption, and the method of transportation to school with the likelihood that youth consumed any breakfast (vs. no breakfast). In addition to the univariate models, two multivariate models examining number of breakfasts consumed and dichotomous breakfast consumption were run that included all the variables listed above as predictors. Results were similar and are not shown. The curvilinear relation between the number of breakfasts consumed and BMI percentile/BMI z-score was examined by adding a quadratic term for number of breakfasts. Race and grade were contrast coded such that each category was compared to all others. Students that reported only drinking water were not counted as having breakfast, but water consumption was examined when identifying what types of foods and drinks students consumed in the morning.

## Results

### Demographics

Sample demographics are shown in Table 1. Most youth were African American (61.4%), 40.5% were overweight or obese (BMI ≥ 85^th^ percentile), including 9.5% who were severely obese (BMI ≥120% of the 95^th^ percentile).

**Table 1:** Socio-demographic characteristics of study participants (N = 651)

**Variable**	**%**
Gender	
Female	52.3
Male	47.7
Race/Ethnicity	
Black	61.4
Hispanic	14.4
Asian	13.2
White	6.9
Other	4.1
Weight Status	
Underweight	2.9
Healthy Weight	56.6
Overweight	15.8
Obese	15.2
Severely obese	9.5
Transportation	
Walked to school	66.0
Driven to school	24.9
Other	9.1
Consumed dinner the previous night	96.1
	*Mean + SD*
Age (yrs)	10.7 ± 1.0
Weight (kg)	44.8 ± 15.1
Height (cm)	146.4 ± 9.2
BMI (kg/m^2^)	20.5 ± 5.1
BMI z-score )	0.7 ± 1.2
BMI percentile	66.6 ± 30.5

Note: SD = standard deviation.

### Breakfast practices

**Figure 1:**
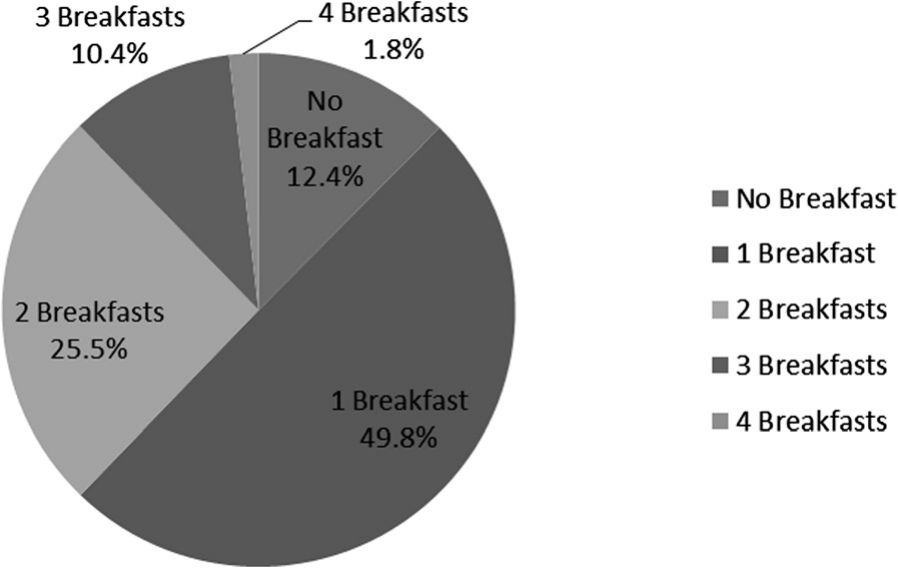
**Proportion of Students Reporting Eating Breakfast (N = 651).** Note: Breakfast was defined as having consumed any caloric food or beverage at any of four specified locations: home, corner store, school cafeteria, and school classroom. All three schools provided all students with access to free breakfast before school hours.

The frequency of breakfast consumption is reported in Figure 1. About half (49.8%) of participants consumed one breakfast. Almost 40% (37.8%) consumed multiple breakfasts: 25.5% consumed two breakfasts and 12.2% ate three or four breakfasts. More than 10% (12.4%) of children had nothing to eat on the morning of survey administration. Among those who did not eat breakfast, reasons included not having time (47.5%), not being hungry (36.7%), disliking available foods (10.8%), not having money to buy food (3.3%), and not having food (1.7%). The proportion of students who reported only consuming beverages at any point during the morning was low (3.4%) and was comparable across home (6.6%), school (1.4%), and corner store (2.4%) locations. The average number of items endorsed in each location was similar (3.7 ± 2.1 items at home, 2.8 ± 1.1 items at school, 2.9 ± 1.3 items at corner store), and it was significantly lower in youth who reported one breakfast compared to youth who reported consuming more than one breakfast (2.19 vs 4.73, t = −11.23, p < .01).

Race, grade, sex, previous night’s dinner consumption, and method of transportation to school were not significantly associated with the number of breakfasts consumed. Youth in 6^th^ grade showed significantly lower odds of any breakfast consumption compared to 4^th^ and 5^th^ graders (OR = 0.46, 95% CI 0.24-0.85). Race/ethnicity, sex, previous night’s dinner consumption, and method of transportation to school were not significantly associated with the odds of breakfast consumption.

### Breakfast practices and relative weight

**Figure 2:**
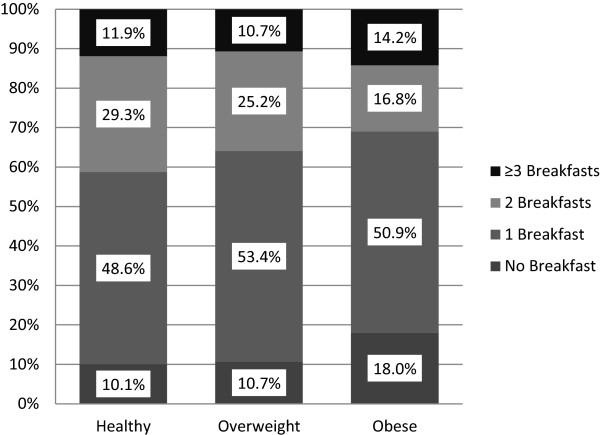
Breakfast frequency by weight status category.

**Figure 3:**
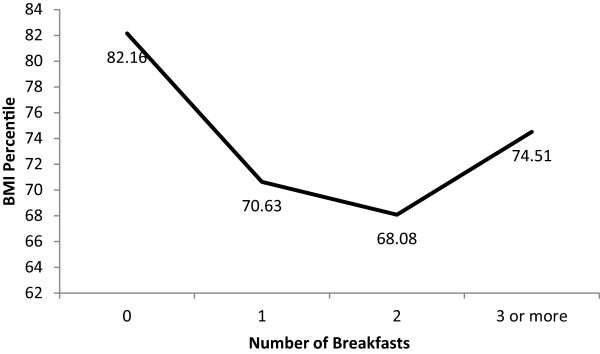
**Model estimated curvilinear relation between BMI percentile and number of breakfasts consumed.** Note: Breakfast was defined as having consumed any caloric food or beverage at any of four specified locations: home, corner store, school cafeteria, and school classroom. Number of breakfasts refers to the number of locations students reported eating breakfast at. All three schools provided all students with access to free breakfast before school hours.

**Figure 4:**
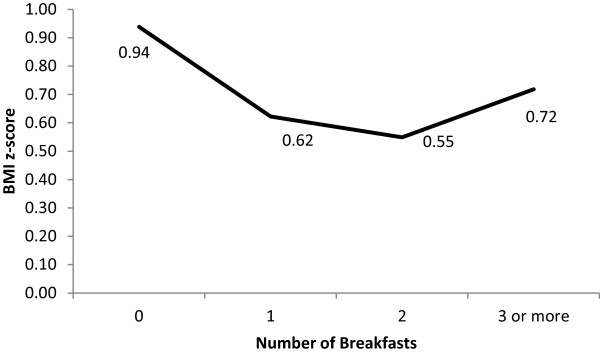
**Model estimated curvilinear relation between BMI z-score and number of breakfasts consumed.** Note: Breakfast was defined as having consumed any caloric food or beverage at any of four specified locations: home, corner store, school cafeteria, and school classroom. Number of breakfasts refers to the number of locations students reported eating breakfast at. All three schools provided all students with access to free breakfast before school hours.

When examining the frequency of breakfast consumption by weight status, obese youth consumed significantly fewer breakfasts compared to healthy weight youth (1.13 vs 1.31, p < .01) and were more likely to report not eating any breakfast compared to healthy weight and overweight youth (18.0% vs. 10.1% and 10.7%, respectively) (Figure 2). No other significant associations were observed between weight status category and the number of breakfasts consumed. When examining associations between continuous BMI percentile and number of breakfasts, there was a curvilinear relationship (B = 4.49, SE = 1.17, p < .01; Figure 3), such that higher mean BMI percentiles were observed among children who did not consume any breakfast and those who consumed ≥ 3 breakfasts (Figure 3). The same curvilinear relationship was shown using BMI z-score (B = 0.12, SE = 0.05, p < .05; Figure 4).

### Breakfast location

Table 2 shows the frequency of breakfast items obtained across locations (i.e., overall, home, corner store, school). The categories of school cafeteria and classroom were collapsed as both categories provided information about foods eaten at school (n = 92 ate in classroom, n = 145 ate in cafeteria). Among those who ate something in the morning, 75.6% ate at home, 32.9% at school and 27.5% at a corner store.

**Table 2:** Students’ consumption of breakfast item categories by location (N = 651)

**Category**	**Overall**	**Home**	**School**	**Corner store**
Ate at any location	87.6%	75.6%	32.9%	27.5%
Cereal	32.5%	37.8%	13.1%	4.5%
Milk, yogurt, or cheese	31.7%	31.7%	25.2%	6.7%
Water	29.0%	27.0%	4.2%	7.8%
100% fruit juice (Juicy Juice)	27.5%	17.5%	36.9%	16.2%
Bread (bagel, toast, or roll)	21.0%	18.1%	22.4%	2.8%
Muffin, donut, pastry, cake, or pie	18.9%	8.9%	28.0%	12.8%
Waffles, French toast, pancakes	17.9%	18.5%	13.1%	1.7%
Breakfast sandwich	17.6%	16.1%	5.1%	15.6%
Chips (Doritos, potato chips, Cheetos, etc.)	17.5%	6.3%	7.0%	44.7%
Eggs	15.3%	18.5%	1.9%	5.0%
Fruits (apple, pear, peaches, etc.)	14.6%	14.8%	7.0%	5.0%
Meat (bacon, ham, sausage), chicken, or fish	13.7%	14.4%	2.8%	8.4%
Soda, lemonade, Capri Sun, Sunny D, Hug, etc.	12.6%	10.0%	1.4%	17.9%
Candy	11.7%	5.3%	4.7%	26.3%
Coffee, tea, iced tea (Arizona, Brisk, etc.)	10.7%	9.4%	2.3%	11.2%
Vegetables (lettuce, green beans, broccoli, etc.)	5.7%	6.1%	4.2%	0.6%
Other	4.3%	2.6%	2.3%	1.1%
Cracker	1.2%	0.8%	2.3%	NA
Pretzel	0.2%	NA	NA	0.6%

Note: Ate at any location indicates the proportion of the sample that endorsed eating breakfast in that location. All three schools provided all students with access to free breakfast before school hours. Category descriptions are presented as they were shown to participants.

Table 3 displays the frequency of breakfast locations and location combinations. Among participants who ate breakfast, they were most likely to eat at home (39.8%) while 8.0% only ate breakfast at school. Among those who had multiple breakfasts, home and school (23.5%) and home and the corner store (23.5%) were the most popular combinations.

**Table 3:** Frequency of breakfast consumption locations (n = 651)

**Variable**	**Count**	**Percent**
Did not eat	81	12.4
Ate Only at Home	259	39.8
Ate Only at School	52	8.0
Ate Only at Corner Store	17	2.6
Ate at Home & School	80	12.3
Ate at Home & Corner Store	80	12.3
Ate at School & Corner Store	9	1.4
Ate at Home, School, Corner Store	73	11.2

Note: All three schools provided all students with access to free breakfast before school hours.

### Food choices

The top five most frequently endorsed food and beverage categories consumed across all locations were cereal, milk, water, 100% fruit juice, bread, and muffin/donut (Table 2). The most common items consumed from home were cereal, milk/yogurt/cheese, water, waffles/pancakes, and eggs. The most common items consumed from school included 100% fruit juice, milk/yogurt/cheese, and muffin/donut. The most common items consumed from corner stores included chips, candy, and soda.

## Discussion

The current study assessed breakfast patterns and relative weight among low-income, 4^th^-6^th^ graders in an urban, low-income area. There were several principal findings.

First, a large number of youth (37.8%) reported eating multiple breakfasts (25.5% consumed 2, and 12.3% consumed 3 or 4). The percent of youth who reported more than 1 breakfast was slightly higher (37.8% vs 30.0%) than what was observed among 3^rd^-5^th^ graders in the New York City schools where universal free school breakfast was similarly provided [10]. Results also showed that youth who had only one breakfast ate significantly fewer items than those who consumed more than one breakfast (2.2 vs 4.7 items), and that youth were eating 3–4 items, on average, at each location, regardless of how many locations at which they ate. This suggests that youth who consumed multiple breakfasts were not simply spreading out the same number of items across locations or consuming a small number of items in each location. These patterns are consistent with those observed among students in New York City in which children who ate at one location reported eating 2.4 food items and children who ate at two or more locations reported eating 5.3 items. Further, the analyses conducted among school children in NYC revealed that a significantly greater number of calories were consumed by youth who ate breakfast at multiple locations [10]. A previous study’s results suggested that increased energy intake at breakfast was sustained throughout the day in elementary school children [11]. It is also important to note that even among healthy weight youth, approximately 12% consumed 3 or more breakfasts (which was greater than the percentage of overweight youth consuming 3 or more breakfasts). Thus, it is important that future research further characterize portion sizes and energy intake in youth consuming multiple breakfasts. Combined, these findings suggest that eating breakfast at multiple locations may contribute to excess energy intake in some youth and that efforts to promote school breakfast consumption may have unintended effects on childhood obesity among low-income elementary school children.

Second, many youth eat breakfast before coming to school. Greater than 75% of youth reported eating breakfast at home, and most of those youth (58%) also ate at school (23.5%), a corner store (23.5%) or both (11.2%). This is similar to New York City youth among whom 69.7% reported eating at home, 20.3% reported eating at a corner store and 30.9% reported eating at school [10]. This trend of consuming multiple breakfasts may be related to the rise in snacking observed in US children [20]. Regardless of whether multiple eating occasions are considered breakfasts or snacks, these findings suggest that interventions designed to increase school breakfast participation should consider that the provided breakfast may be in addition to what was already consumed prior to school at home and corner stores. These data suggest it is important to communicate to parents about the opportunities for youth to eat breakfast (at both corner stores and school). Such knowledge can inform parent’s advice to their children about breakfast options and may influence what is served at home for breakfast. The higher nutritional quality of foods reported at school and home breakfasts is desirable due to its connection with learning and behavioral outcomes [1]. However, the low nutritional quality of foods consumed by elementary school children at corner stores has implications for excess energy intake and obesity [19]. Specifically, the most frequently purchased foods in corner stores (chips, candy, and soda) are high in solid fats and added sugars, which are excessive among US children [21].

The third principal finding was that 12.4% of youth reported not eating breakfast despite the free breakfast being offered at school. Other estimates for youth not eating breakfast have ranged from 10%-30% [1]. The top reasons youth provided in the current study (i.e., lack of time, not feeling hungry) are consistent with those provided in other studies [1]. It has been suggested that novel school breakfast strategies, such as classroom breakfast, are needed to adequately provide breakfast to food-insecure children [4]. However, in the current study there were only 6 reports of children not eating breakfast due to a lack of resources, although validated measures of household food security were not employed. More research is needed to determine how school breakfast policies may be able to address breakfast intake among food insecure students.

The fourth principal finding was that obese youth were more likely to have no breakfast (18.0%) as compared to healthy (10.1%) and overweight (10.7%) youth when examining breakfast consumption dichotomously. In addition, when examining breakfast consumption continuously (0 - ≥3 breakfasts), there was a significant curvilinear relation between BMI percentile and number of breakfasts consumed, with higher mean BMI percentiles among children who did not consume any breakfast and those who consumed ≥ 3 breakfasts. This non-linear relation between relative weight and breakfast consumption may partially explain why previous studies in elementary school children have at times failed to show a bivariate (i.e., linear) relation between BMI percentile and breakfast consumption [11,22]. Previous research has supported the role of breakfast consumption for reducing later energy intake in a small sample of normal to overweight breakfast skipping adolescents [23], but the association between relative weight and breakfast consumption among elementary, middle, and high school children has mixed results in previous studies [1,8,24]. Some studies found that breakfast was associated with a lower relative weight [25-27] while others did not [11,22]. Given the lack of information on portions and energy intake in the current study, the slight and non-significant differences in BMI percentile with 1 versus 2 breakfasts consumed should be interpreted with caution. Even so, the finding of higher mean BMI percentiles among children who did not consume any breakfast and those who consumed ≥ 3 breakfasts suggests the need for careful study of breakfast policies, such as breakfast in the classroom, that offer all youth (additional) opportunities to eat breakfast. These data do not suggest that school breakfast feeding should be abandoned. They do, however, suggest that careful attention be paid to policies around breakfast and that any decisions to change policies should be carefully considered with appropriate data.

The study had important strengths including measured height and weight, assessment of breakfast at and outside of school, and inclusion of a low-income, minority sample. The higher rates of overweight/obesity and severe obesity in the current study compared to nationally representative data underscore the importance of carefully studying health policies that may have impacts on health disparities. Furthermore, it is possible that opportunities for assessing multiple breakfasts differ across urban and rural or suburban settings or across locations with sparse access to food outlets, and future studies are needed to characterize multiple breakfasts in nationally representative samples. The breakfast questionnaire’s strength includes a relatively quick recall of foods from a few hours earlier that morning. Its weaknesses include the measurement of only a single day and the lack of a full day of intake. The lack of dietary information for the full day also raises a question of potential compensation over the remainder of the day. However, another previously described study showed that elementary school children who ate 2 or more breakfasts reported consistently higher energy intakes throughout the day [11]. The current study was not able to precisely quantify energy intake because data regarding quantities, portion sizes, or nutrient content of food (e.g., milk not described as whole, low-fat, non-fat/skim) were not assessed. It is possible that at each of the multiple breakfasts children did not consume the entire portion. It is also possible that individuals who obtained breakfast foods from multiple locations may not have consumed multiple “full” breakfasts, and thus, the number of breakfasts may be overestimated. It is clear, however, that children who had multiple breakfasts consumed more total items and approximately the same number of items at each location, which would contribute to increased intake. Data from the current study relate to where breakfast was obtained rather than where it was ultimately consumed. Anecdotal evidence suggests it was most often consumed where it was obtained, but the inability to directly observe where breakfast items were obtained versus consumed precludes definitive conclusions. Additionally, the study is cross-sectional, so the direction of the relations between BMI percentile, relative weight, and breakfast patterns cannot be determined.

## Conclusion

In conclusion, few studies have assessed breakfast consumption patterns both at and outside of school in among youth. This study is the first to also examine measured height and weight and multiple breakfasts in youth. The current data found almost 40% of low-income urban youth consumed multiple breakfasts while 12% consumed none. When examining breakfast dichotomously (i.e., breakfast, no breakfast), a significantly higher percent of obese youth did not eat breakfast compared to healthy and overweight youth. When examining number of breakfasts (i.e., 0 - ≥3 breakfasts), higher mean BMI percentiles were observed among children who did not consume any breakfast and those who consumed ≥ 3 breakfasts. These findings may have implications for school breakfast policies, childhood obesity, family communications about breakfast and food insecurity. These data indicate there may be unintended consequences of providing multiple opportunities for elementary school children to eat breakfast. Equally concerning is the fact that current school breakfast policies are still leaving 12.4% of youth without breakfast. Continued efforts to address both childhood obesity and food insecurity are needed.

## Abbreviations

SBP: School breakfast program; BMI: Body mass index.

## Competing interests

The authors declare that they have no competing interests.

## Authors’ contributions

HGL, HP, MA, SS, JOF, KWB, LBD, JN, GVW and GDF designed research; SVV, HP, MA, SS, and TS conducted research; HGL and TS analyzed data; HGL, KWB, JOF, LBD and GDF wrote the paper; HGL had primary responsibility for the final content; all authors read, made revisions and approved the final manuscript.

## Authors’ information

JN is now at Olin Neuropsychiatry Research Center, Institute of Living.

GDF is now employed by Weight Watchers International.

## Pre-publication history

The pre-publication history for this paper can be accessed here:

http://www.biomedcentral.com/1471-2458/14/604/prepub
